# Bariatric Surgery: An Opportunity to Improve Quality of Life and Healthy Habits

**DOI:** 10.3390/nu16101466

**Published:** 2024-05-13

**Authors:** Beatriz Vanessa Díaz-González, Inmaculada Bautista-Castaño, Elisabeth Hernández García, Judith Cornejo Torre, Juan Ramón Hernández Hernández, Lluis Serra-Majem

**Affiliations:** 1 Triana Primary Health Care Center, Canarian Health Service, 35002 Las Palmas de Gran Canaria, Spain; beatrizvanessa@gmail.com; 2 Research Institute of Biomedical and Health Sciences (IUIBS), University of Las Palmas de Gran Canaria, 35016 Las Palmas de Gran Canaria, Spain; elisabethernandez924@gmail.com (E.H.G.); judith.cornejo.dn@gmail.com (J.C.T.); jurahh@yahoo.es (J.R.H.H.); lluis.serra@ulpgc.es (L.S.-M.); 3 Centro de Investigación Biomédica en Red Fisiopatología de la Obesidad y la Nutrición (CIBEROBN), Instituto de Salud Carlos III, 28029 Madrid, Spain; 4 Centro Hospitalario Universitario Insular Materno Infantil (CHUIMI), Canarian Health Service, 35016 Las Palmas de Gran Canaria, Spain

**Keywords:** morbid obesity, bariatric surgery, Mediterranean diet, health-related quality of life, physical activity, depression

## Abstract

Bariatric surgery therapy (BST) is an effective treatment for obesity; however, little is known about its impacts on health-related quality of life (HRQoL) and related factors. This study aimed to evaluate changes in HRQoL and its relationship with weight loss, depression status, physical activity (PA), and nutritional habits after BST. Data were obtained before and 18 months postprocedure from 56 obese patients who underwent BST. We administered four questionnaires: Short Form-36 health survey for HRQoL, 14-item MedDiet adherence questionnaire, Rapid Assessment of PA (RAPA) questionnaire, and Beck’s Depression Inventory-II. Multivariable linear regression analysis was used to identify factors associated with improvement in HRQoL. After the surgery, MedDiet adherence and HRQoL improved significantly, especially in the physical component. No changes in PA were found. Patients without previous depression have better mental quality of life, and patients who lost more than 25% of %TBWL have better results in physical and mental quality of life. In the multivariable analysis, we found that %TBWL and initial PCS (inversely) were related to the improvement in PCS and initial MCS (inversely) with the MCS change. In conclusion, BST is an effective intervention for obesity, resulting in significant weight loss and improvements in HRQoL and nutritional habits.

## 1. Introduction

Obesity, defined as a body mass index (BMI) ≥ 30 kg/m^2^, is a significant public health problem, with its worldwide prevalence growing considerably, tripling between 1975 and 2016 [1]. It is recognized as a disease by the American Medical Association [2], the World Health Organization [1], and the World Obesity Federation [3], and it is a predisposing factor for various pathologies, such as type 2 diabetes mellitus, hypertension, cardiovascular diseases, different cancers, and premature death [4,5].

Severe or morbid obesity, defined as a BMI > 40 (obesity class III), presents a significant therapeutic challenge, as nutritional and pharmacological interventions often prove ineffective in the long term [6].

BST emerges as the most effective treatment option, demonstrating favorable outcomes in terms of weight loss and sustained maintenance [7]. The two most common procedures used currently, the sleeve gastrectomy and gastric bypass, have similar effects on weight loss and diabetes outcomes and similar safety through at least 5-year follow-up. However, emerging evidence suggests that the sleeve procedure is associated with fewer reoperations, and the bypass procedure may lead to more durable weight loss and glycemic control. Different types of bariatric surgery have distinct auxiliary and adverse effects, emphasizing the importance of individualized selection of procedures and risk–benefit conversations for each patient considering bariatric surgery [8,9].

The current data indicate that the perioperative mortality rates of BST range from 0.03% to 0.2%, which has substantially improved since the early 2000s [8,9]. A recent review of 66 health outcomes of bariatric surgery has shown that 56 outcomes were health benefits, including new-onset of diabetes, dyslipidemia, cardiovascular diseases, hypertension, cancers, women’s health, and reductions in all-cause mortality and 10 were adverse outcomes, including suicide, fracture, gastroesophageal reflux after sleeve gastrectomy, and neonatal morbidities, but none of the adverse outcomes reached a high level of evidence, warranting the need for further investigation.

In addition to the several pathologies mentioned above, obesity is associated with deterioration in the perceived quality of life, especially its physical component score [10,11,12,13]. Mobility problems, reduced self-care, a decreased ability to carry out daily activities, pain, and discomfort are some of the problems suffered by people with obesity. Some characteristics of this pathology prevent patients from making the changes to vital habits that are necessary to promote and maintain a good quality of life, and this is the reason why another widely used outcome measure in obesity treatment is the quality of life and changes thereof [14].

Improvements in health-related quality of life (HRQoL) obtained by weight loss have been reported after BST [15], bariatric endoscopy treatment [16], or conventional treatment of obesity [17]. The impact of bariatric surgery on HRQoL is less well-understood than its clinical effectiveness in terms of weight and comorbidities. Some studies suggest that the physical aspects of HRQoL may improve more than the mental health aspects of HRQoL after bariatric surgery. Due to psychological predispositions, some patients appear to be less likely to benefit from bariatric treatment, whether in terms of HRQOL [10,11].

Moreover obesity is associated with deteriorated quality of life, and there exists a well-known bidirectional relationship between obesity and depression [18,19]. When analyzing various levels of obesity (class I: BMI > 30–<34.9, class II: BMI >35–<39.9, class III: BMI > 40), it seems that the degree of obesity consistently correlates with an increased risk of depression [20,21]. The early identification of such patients and providing them with psychological intervention would likely further improve the outcomes of bariatric treatment.

During the BST follow-up, it is very important to make changes to nutritional and PA habits to improve health and maintain a healthy weight. Regarding nutritional habits, the Mediterranean diet (MedDiet) is suggested as a healthy dietary pattern, consistently demonstrating a positive impact on health and longevity. The MedDiet includes lots of healthy foods like olive oil as the main culinary fat, whole grains, fruits, vegetables, fish, seafood, beans, and nuts, and a low intake of red meat and sweets [22,23].

Several studies have indicated that the MedDiet is not only a preventive measure against cardiovascular and other diseases but could also be highly effective in terms of weight loss, particularly when combined with energy restriction and increased PA [23,24]. Different studies, like the SUN project [25] and the Predimed plus study [26], have shown that in addition to the benefits of cardiovascular risk, higher adherence to the MedDiet pattern is associated with better HRQoL.

Furthermore, PA is recommended as a key component of weight management for preventing weight gain, promoting weight loss, and preventing weight regain after weight loss. PA also yields other positive effects on various health indicators, including overall mortality, cardiovascular disease mortality, incident hypertension, incident type 2 diabetes, incident site-specific cancers, mental health (such as reduced symptoms of anxiety and depression), cognitive health, and sleep [6,7].

Several studies have observed an improvement in quality of life after BST [10,11,12]; however, there is a lack of studies on factors related to previous depression status, eating habits, or PA that influence changes in quality of life post-BST.

This study aimed to evaluate other factors, different from weight loss, that could influence the improvement of HRQoL post-BST.

## 2. Material and Methods

### 2.1. Study Design

We conducted a prospective study involving 56 consecutive patients who underwent BST at Vithas Santa Catalina Hospital in Las Palmas, Spain, between April and July 2015. The BST was offered by the public health service.

Participants were provided with four questionnaires: (a) the SF-36 survey (Spanish version) to measure HRQoL, (b) a 14-item questionnaire assessing adherence to the MedDiet, (c) the Rapid Assessment of Physical Activity (RAPA) questionnaire to measure PA, and (d) Beck’s Depression Inventory (BDI) to assess the depression status. The trial was approved by the Institutional Review Board of the CEIC del Hospital Universitario Insular de Gran Canaria (HUIGC).

### 2.2. Participants

We invited 56 obese patients who were referred for BST after a failed diet and lifestyle intervention to participate in the study. All agreed to take part and answered the questionnaires at baseline and 18 months postprocedure. We delivered the questionnaires during the regular follow-up visit.

The weight, height, and body composition were recorded using the Tanita BC-420MA III, Barcelona, Spain, before the intervention and during the follow-up visits. We reported weight loss outcomes as %total body weight loss (%TBWL).

### 2.3. Intervention

Regarding the type of procedure, 64.3% (n = 36) underwent restrictive techniques and 35.7% (n = 20) malabsorptive techniques.After the surgery, patients were regularly monitored by both the surgeon and endocrinologist, who provided guidance to enhance physical activity and adhere to a low-calorie Mediterranean diet.

### 2.4. Questionnaires

At baseline and 18 months after the BST, the patients complete different questionnaires in a face-to-face interview with the research team. All the questionnaires used in the present study had enough reliability and validity and were validated in the European Spanish population (Beck questionnaire for depression [27], RAPA questionnaire for physical activity [28], and SF36 for HRQoL were validated for the Spanish population [29]. The Mediterranean Diet adherence test has also been adequately validated and used in important studies such as the PrediMed study [30].

#### 2.4.1. Short Form Survey-36 (SF-36)

The SF-36 is a well-validated questionnaire for the Spanish population that measures the patients’ self-reported opinions on their physical and mental well-being. It has eight domains of HRQoL: general health, physical functioning, role limitations due to physical health, body pain (functional status), energy/vitality, emotional well-being, role limitations due to emotional problems, and social functioning (emotional status). Responses to each question within a domain are combined to generate a score from 0 to 100, where 100 indicates “good health”. The domains are calibrated and transformed into the physical component summary (PCS) and mental component summary (MCS), respectively. The SF-36 also includes a global health transition question (HTQ) that asks respondents to rate their general health compared with 12 months ago. We compared the results with the reference Spanish population SF-36 outcomes for the study by Lopez Garcia et al., who prospectively evaluated the HRQoL in 6207 Caucasian noninstitutionalized individuals aged > 18 years from Spain [31].

#### 2.4.2. Fourteen-Item Questionnaires That Assessed Adherence to a MedDiet

The adherence to the MedDiet was assessed using the 14-item MedDiet Adherence Screener (MEDAS), a validated questionnaire, which consisted of 2 questions on dietary habits and 12 questions on food consumption frequency characterizing the MedDiet pattern. Each question was scored 0 or 1. A value of 0 was assigned when the condition was not met. The total score ranged from 0 to 14. The adherence level is determined by adding the values obtained from the 14 items. Three levels are established: (a) a total score ≥ 10 indicates high adherence, (b) a score between 7 and 9 (inclusive) indicates a medium adherence level, and (c) a total sum ≥ 6 suggests low adherence to the MedDiet. To simplify the analysis, the results were recoded into two groups: (a) low adherence < 6 and (b) medium–high adherence ≥ 6 points [30].

#### 2.4.3. Rapid Assessment of Physical Activity (RAPA) Questionnaire

To measure the level of PA, the Rapid Assessment of PA Scale (RAPA) [32] questionnaire was used. This particular test was selected due to its simplicity and ease of performance. RAPA is divided into two categories: RAPA 1, which determines the level of PA: (a) sedentary (‘I rarely or never do any physical activities’); (b) little active (‘I do some light or moderate PA, but no every week’ or ‘I do some light PA every week’); (c) moderately active (‘I do moderate PA every week, but less than 30 min a day or 5 days a week’ or ‘I do vigorous physical activities every week, but less than 20 min a day or 3 days a week’); and (d) active (‘I do 30 min or more a day of moderate PA, 5 or more days a week’ or ‘I do 20 min or more a day of vigorous PA, 3 or more days a week’). RAPA 2, which assesses the type of exercise: (a) no muscle strength and flexibility activities; (b) muscle strength activities (‘I do activities to increase muscle strength, such as lifting weights or calisthenics, once a week or more’; (c) flexibility activities (‘I do activities to improve flexibility, such as stretching or yoga, once a week or more’); or (d) both muscle strength and flexibility activities). For further analysis, participants were divided into two groups according to their RAPA 1 score: “inactive” (“sedentary” + “minimal active”) (score 1–3) and “active” (“moderate active” + “active”) (score 4–7).

#### 2.4.4. Beck’s Depression Inventory-II

The depression level was analyzed with the Beck’s Depression Inventory-II (BDI-II) [33]. It is a 21-question (multiple choice) self-report inventory created to provide a quantitative assessment of the severity of depression considering the DSM-IV criteria for diagnosing depressive disorders and includes items measuring cognitive, affective, somatic, and vegetative symptoms of depression. The score scale ranges from 0 to 3 for each question; higher scores indicate more depressive symptoms. Scores of 0–13 are considered to indicate none or minimal depression, 14–19 mild depression, 20–28 moderate depression, and ≥29–63 severe depression. For the purpose of the present study, this variable was simplified as none or minimal depression (scores 0–13) and mild–severe depression (scores ≥ 14).

### 2.5. Outcomes

This study aimed to evaluate changes in HRQoL and its relationship with weight loss, depression status, PA, and nutritional habits after BST.

### 2.6. Statistical Methods

We expressed continuous variables as mean and measures of dispersion (standard deviation; SD). We reported categorical variables as a percentage. For analysis, we categorized the variables based on (a) gender (male/female), (b) age (≤43 or >43 years), (c) initial BMI (<40 or ≥40 kg/m^2^), (d) procedure type (restrictive or malabsorptive techniques), (e) PA status (inactive or minimally active/active), (f) MedDiet adherence level (low adherence ≤ 6 and medium–high adherence > 6 points), (g) depression status (none or minimal and mild–severe), and (h) %TBWL (≤25% vs. 25.1–39.9% vs. ≥40%). Bivariate analyses of proportionality of distribution of categorical variables Chi-square test (χ^2^). For continuous variables, we used the Kolmogorov–Smirnov test to check that the variables were normally distributed. For comparisons of normally distributed variables, we used the T-Student test or ANOVA. For comparisons of non-normally distributed variables, we used the Mann–Whitney or Kruskal–Wallis tests.

We performed multivariable regression analysis to study the relationship between changes in PSC and MSC scores as dependent variables and age, gender, %TBWL, changes in PA, depression status, and initial summary component values as independent variables.

We performed statistical analysis using IBM SPSS Software 23.0. *p* < 0.05 was considered significant.

## 3. Results

### 3.1. Participants Characteristics

The baseline characteristics of the 56 patients are detailed in Table 1. The mean age was 43.8 ± 13.1 years (range 20–69), and most of them were female (n = 41, 73.2%) and with BMI ≥ 40 kg/m^2^ (n = 48, 85.7%).

### 3.2. Weight Loss Outcomes

Regarding the variation in weight parameters, the mean weight loss was 45.2 ± 20.7 kg, the BMI loss was 16.8 ± 7.1 units, and the mean %TBWL was 34.4 ± 13.3. In relation to the surgical technique, a significantly greater weight loss was observed in the malabsorptive compared with restrictive techniques: 54.8 vs. 39.9 kg of weight loss, 19.9 vs. 15.1 units of BMI loss, and 39.6 vs. 31.6% of %TBWL. No significant differences were found by sex with respect to weight loss. By age group, those younger than 43 years lost more weight than those older (50.7 vs. 39.4 kg *p* = 0.41). Finally, in relation to %TBWL, 13 patients (23.2%) lost ≤ 25%, 20 (37.5%) between 25 and 40%, and 21 patients (39.3%) lost ≥ 40%.

### 3.3. Changes in MedDiet Adherence

After the surgery, the mean score improved significantly from 6.32 (1.9) to 7.62 (1.8) (*p* < 0.001). We did not find any significant relationship between the improvement in MedDiet adherence with gender, surgery procedures, or age group.

Considering the level of adherence, at baseline, 60.4% had low, 35.8% medium, and 3.8% had high levels of adherence to the MedDiet. Postintervention, 32.1% had low, 60.4% medium, and 7.5% high adherence to the MedDiet.

Table 2 shows the changes in the MedDiet group adherence prior to and after the intervention. Most of half the patients with low levels of adherence improve their adherence to a medium–high level (*p* = 0.04).

### 3.4. Changes in Physical Activity

Considering the level of PA, for practical purposes, the 54 patients who answered the questionnaire were segregated into two groups: inactive and active. Although the difference was not significant (*p* = 0.09), before the surgery, 79.6% were inactive, and postintervention, only 44.5%. There was also a low improvement in the practice of muscle strength and flexibility exercises.

### 3.5. Changes in Depression

The Beck questionnaire results showed that pre- and postprocedure depression status was very similar, with less percentage of none or minimal depression status postprocedure (Table 2).

### 3.6. Changes in HRQoL

**Figure 1:**
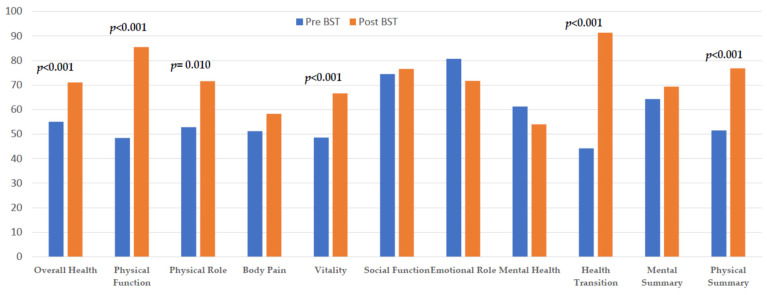
Variation in mean values of SF-36 Health Status components in subjects undergoing bariatric surgery.

The mean baseline PSC score was 51.5 (range 9.5–96.9), and the MSC score was 64.3 (range 1.43–98.6). After the intervention, we found a significant improvement in the PSC score (76.8 vs. 51.5), but we did not find a significant difference in the MSC score. We observed a significant improvement in most of the domains except body pain, social function, emotional role, and mental health domain of the HRQoL. The domains that demonstrated the greatest and most significant improvement were physical function, physical role, vitality, overall health, and health transition (Figure 1 and Table 3).

If we compared the score values of the obese patients with the Spanish population values (Table 3), all of the domains were lower prior to the intervention. Postprocedure, overall health was higher, and physical function and vitality were very similar. The emotional status, except vitality, continues with lower values than the Spanish population’s mean values.

When stratified by the baseline characteristics of the PSC and MSC scores, we observed higher PSC initial levels in patients with younger ages (<43 years) and no or minimal depression. The baseline MSC score was better in patients with no or minimal depression. The final MSC score was better in patients with %TBWL 25–40% and in patients with low or minimal depression.

In relation to changes in physical and mental summary scores, we noticed significantly higher PSC score change in those patients who achieved greater %TBWL (>25%). The change in MSC score only results in significance in older patients (≥43 years) and previously inactive patients (Table 4).

We did not find any significative difference in relation to changes in PSC and MSC scores and depression levels, but in the MSC change, the high depression group had better improvement (Table 4).

### 3.7. Predictive Factors for Improvement in PSC and MSC

In the linear regression analysis, considering the change in the PSC as the dependent variable and the %TBWL, age and sex, initial PSC, and depression status as independent variables, we found that the %TBWL was significantly related to the improvement in the PSC (*p* < 0.015) and the initial PSC was inversely related to this improvement (baseline *p* < 0.001). In the case of the mental summary, considering the change in the MSC as the dependent variable and the %TBWL, age and sex, initial MSC, and depression status as independent variables, we found that the change in this summary was only inversely associated with the initial mental summary (*p* < 0.001) (Table 5).

## 4. Discussion

The current study demonstrates that following BST, adherence to the MedDiet and HRQoL significantly improved, particularly in the physical component. Additionally, patients without a history of depression and those who achieved a weight loss exceeding 25% of total body weight loss (%TBWL) experienced notably better outcomes in both physical and mental quality of life. However, we did not observe significant changes in PA [34,35,36].

Regarding HRQoL in the obese population, some authors have found that obesity has negative impacts, particularly affecting physical domains (e.g., physical function, body pain, overall health) and the physical component summary (PCS) of the SF-36 [12,37]. Poor emotional well-being among the obese may be attributed to comorbidity rather than obesity per se [38]. If we compare the initial HRQoL with the general population, our results confirm that the obese population perceives they have a worse quality of life than the non-obese population [31].

BST has consistently demonstrated long-term significant weight loss and a notable improvement in medical comorbidities [39]. Additionally it has been associated with lower rates and fewer symptoms of various mental health conditions, particularly depression, which improved following the intervention [40,41,42]. However, we did not find improvement in depression levels at 18 months in the present study, which remained similar to the baseline. On the other side, our study aligns with some studies that found higher preoperative depression severity as a predictor of poorer improvement in quality of life change after BST [43,44]. However, different authors have not found clear evidence that preoperative mental health conditions are associated with differential weight loss after surgery [35,45]. In fact, some studies reported that preoperative psychopathology predicted post-operative psychopathology but not weight loss at 2 years [40,46].

Considering the often inversely proportional relationship between weight and self-perceived health [38], weight loss obtained by the BST should improve quality of life. Several authors have suggested a linear correlation between weight changes and HRQoL [47,48]. Studies evaluating HRQoL after BST have demonstrated a favorable change in both physical and mental health at 3 and 6 years and an improvement in PA [49,50]. However, when weight loss is moderate, different authors have not shown improvement in the domains of the SF-36 [51].

Regarding BST, most published trials have found an improvement in HRQoL in physical and mental summary components (SF-36) [51,52]. We found that the best changes in PSC were obtained with better results in weight loss, especially in patients with more than 25% of %total body weight loss (%TBWL). This replicates the findings from Kolotkinn et al., who also found that after a weight loss of 34.2% after gastric bypass surgery, patients experienced significant improvement in HRQoL [53]. Other studies obtained the same results [54].

The mental component summary (MSC), however, did not improve with the intervention, but older and inactive patients prior to the intervention exhibited better positive changes in MSC. Initial MSC was inversely associated with better changes in mental summary postprocedure. Some authors have reported an initial improvement in this sphere and subsequent deterioration at the end of the follow-up period [55]. However, results obtained by Paczkowska et al. have shown that the quality of mental health among patients with morbid obesity significantly depends on gender and the percentage of total weight loss achieved as a result of treatment [56]. Most scientific data have shown that improvement in a patient’s mental health is likely attributed to weight loss and resultant gains relating to body image, self-esteem, and self-concept [57].

Our results are similar to those obtained by Brunault et al., with better improvement in physical HRQoL in patients who had higher weight loss and lower preoperative depression severity but are different in the better results in younger patients [44]. We found better results in patients older than 43.

After undergoing BST, patients exhibit enhanced adherence to the MedDiet. However, we did not find a positive association between higher adherence to a MedDiet or better PA habits and better quality of life, as found in other studies [26]. We believe that further studies with longer follow-up times are needed in order to clarify this question.

As for the limitations of the study, we found that the sample size was not very large, and patients with morbid obesity stem from a complex psychological sphere and have lower perceived health across all dimensions of quality of life. While it is true that the results have been assessed at 18 months post-intervention, it is possible that this improvement in the quality of life perceived by the patient is not maintained over time. Therefore, it would be advisable to reevaluate the patients 5 and 10 years after the intervention, considering that there are studies that report an initial improvement that is not sustained over time but is still better than before the intervention [58,59,60].

On the other hand, the use of SF-36 provides useful information, but it does not contemplate specific problems from obesity, such as body image and social stigma [61], that are better measured by the IWQoL-Lite instrument, developed for use in people with morbid obesity [62], or the OR-WELL-R, a new obesity-related quality-of-life instrument for assessing the “individual experience of overweightness” [63].

Preoperative mental health assessment and management are critical components of comprehensive care in the BST process. Identifying and addressing mental health issues prior to surgery can contribute to more successful outcomes and improved long-term quality of life for patients. It is critical that health care professionals work collaboratively with patients to address these issues and provide comprehensive support.

Health professionals should approach depression cautiously in their obese patients, and in certain instances, addressing this mental health condition might be the first step in managing them.

## 5. Conclusions

BST is an effective intervention for obesity, resulting in significant weight loss, improvements in HRQoL, and better nutritional habits. Patients without previous depression have better initial and final mental quality of life, and patients with optimal clinical response in weight loss have better results in physical and mental quality of life changes.

It is advisable for all members of the multidisciplinary team responsible for selecting and monitoring patients undergoing bariatric surgery to carefully select the most suitable candidates to benefit from this procedure. Additionally, close monitoring of patients, particularly providing psychological support to those with poorer mental health, is crucial. Well-designed studies with large sample sizes are needed to investigate factors other than weight loss that may be related to improvements in the physical and mental health of patients following bariatric surgery techniques.

## Figures and Tables

**Table 1:** Characteristics of study participants at baseline.

Variables	Patients (n = 56)
Ages years (mean (SD)	43.8 (13.1)
Age groups	
≤43 years (n (%))	29 (51.8%)
>43 years (n (%))	27 (48.2%)
Female	41 (73.2%)
Initial weight kg (mean (SD)	128.9 (23.5)
Initial BMI kg/m^2^ (mean (SD)	47.7 (6.4)
Initial BMI group	
35–39.9 (n (%))	8 (14.3%)
≥40 (n (%))	48 (85.7%)

**Table 2:** Changes in MedDiet adherence, physical activity, and depression after bariatric surgery.

Variable	Preprocedure	Postprocedure	*p*
MedDiet adherence group (n (%))			
Low	32 (60.4)	17 (32.1%)	0.04
Medium	19 (35.8)	32 (60.4%)	
High	2 (3.8)	4 (7.5%)	
Physical activity level aerobic			
Inactive	43 (79.6)	24 (44.5)	NS
Active	11 (20.4)	30 (55.5)	
Physical activity level anaerobic			
None	51 (94.4)	45 (84.9)	NS
Muscle strength	3 (5.6)	6 (11.3)	
Flexibility	0	2 (3.8)	
Depression			
Minimal	36 (67.9)	38 (71.7)	NS
Mild–severe	17 (32.1)	15 (28.3)	

**Table 3:** Variation in mean values of SF-36 Health Status components in a subject undergoing bariatric surgery. Comparison with population values [29].

SF-36 Domains		Spanish Population Mean	Preprocedure Mean	Postprocedure Mean	Mean Difference	*p*
Functional Status	Overall health	58.3	55.0	71.1	14.2	<0.001
	Physical function	84.7	48.4	85.5	37.3	<0.001
	Physical role	83.2	52.8	71.6	18.6	0.010
	Body pain	79.0	51.2	58.3	7.5	NS
Emotional Status	Vitality	66.9	48.6	66.6	17.3	<0.001
	Social function	90.1	74.5	76.6	0.98	NS
	Emotional role	86.6	80.7	71.7	−7.1	NS
	Mental health	73.3	61.2	54.0	−6.8	0.051
Health transition		---	44.2	91.3	52.4	<0.001
MCS (mental component summary)			64.3	69.3	5.8	NS
PCS (physical component summary)			51.5	76.8	25.1	<0.001

**Table 4:** Baseline, final, and variations in physical and mental scores of SF-36 according to selected variables in subjects undergoing bariatric surgery.

	Physical Summary Component (Mean)			Mental Summary Component (Mean)		
	Baseline	Follow-Up	Δ Change	Baseline	Follow-Up	Δ Change
Age						
<43 years	59.6	78.1	18.1	68.9	66	−0.82
≥43 years	42.7	75.6	33.1	59.3	73	13.8
*p*-value	0.010	NS	NS	NS	NS	0.045
Gender						
Male	61.1	79.2	17.4	68	69.3	1.2
Female	48.1	76.1	27.9	63.1	69.4	7.6
*p*-value	NS	NS	NS	NS	NS	NS
Initial BMI						
<40 kg/m^2^	57.4	64.8	7.3	70.1	60.7	−1.3
≥40 kg/m^2^	50.5	78.8	28.0	63.6	70.7	6.9
*p*-value	NS	NS	NS	NS	NS	NS
%TBWL						
≤25	56.1	62.2	6.1	56.6	57.9	1.3
25.1–39.9	53.3	84.0	30.8	68.4	83.0	16.5
≥40	47.4	77.8	30.3	65.2	62.3	−0.7
*p*-value	NS	0.011	0.020	NS	0.001	NS
Procedure Type						
Restrictive	54.5	78.6	25.8	66.4	72.6	8.4
Malabsortive	49.7	73.8	23.8	60.1	63.4	1.3
*p*-value	NS	NS	NS	NS	NS	NS
Mediterranean Diet Adherence n (%)						
Low	52.6	77.6	24.2	62.1	69.1	9.0
Medium–High	50.1	75.9	26.4	67.7	69.9	1.1
*p*-value	NS	NS	NS	NS	NS	NS
Baseline RAPA Aerobic activities n (%)						
Inactive	48.9	77.4	28.1	62.7	71.7	10.2
Active	61.7	75.0	13.1	70.9	60.8	−10.6
*p*-value	NS	NS	NS	NS	NS	0.019
Depression (Beck)						
None or Minimal	58.9	79.5	20.3	73.7	75.0	1.35
Mild–Severe	36.9	70.3	35.0	45.1	57.0	15.8
*p*-value	0.001	NS	NS	<0.001	0.041	NS

**Table 5:** Factors associated with improvement in physical and mental summary components of SF-36 (multivariable linear regression analysis).

Variable	Physical HRQoL (PSC Change)	95% CI	*p*-Value
	Coefficient β		
%TBWL	0.313	0.126 to 1.151	0.015
Sex	0.043	−10.574 to 15.866	0.689
Age	0.009	−0.565 to 0.602	0.949
Initial PSC score	−0.697	−1.144 to 0.447	0.000
Initial Depression	−0.167	−23.397 to 4267	0.170
Variable	Mental HRQoL (MSC change)	95% CI	*p*-value
	Coefficient β		
%TBWL	0.106	−0.359 to 0.751	0.480
Sex	−0.043	−17.804 to 11.985	0.737
Age	0.114	−0.315 to 0.758	0.409
Initial MSC score	−0.677	−1.135 to −0.382	0.000
Initial Depression	−0.191	−29,249 to 8.566	0.246

HRQoL = health-related quality of life; SF-36 = Short Form-36 health survey for HRQoL; PSC = physical component summary; MSC: mental component summary; %TBWL = %total body weight loss.

## Data Availability

The data presented in this study are available on request from the corresponding author due to ethical reasons.
